# Supplementary material for: Maternal Vegetable and Fruit Consumption during Pregnancy and Its Effects on Infant Gut Microbiome
Source: Nutrients. 2021 May 5;13(5):1559. doi: 10.3390/nu13051559 (PMC8148194; doi:10.3390/nu13051559)
Supplement: Supplementary file 1 [file nutrients-13-01559-s001.zip › nutrients-1165483-supplementary.pdf]

Maternal Vegetable and Fruit Consumption During Pregnancy and Its Effects on Infant Gut Microbiome

Hsien-Yu Fan, Yu-Tang Tung, Yu-Chen Yang, Justin BoKai Hsu, Cheng-Yang Lee,

Tzu-Hao Chang, Chia-Yu Su, Rong-Hong Hsieh, Yang Ching Chen

Supplemental figures

Figure S1. Flowchart of participant recruitment............1

Figure S2. Alpha diversity of the infant gut microbiome according to maternal vegetable and fruit consumption during pregnancy............2

Figure S3. Variations in the infant gut microbiome are not explained by other potential confounders............3

Figure S4. Relationship of intake of food from specific food groups to the abundance of specific gut microbiota............5

Figure S1. Flowchart of participant recruitment

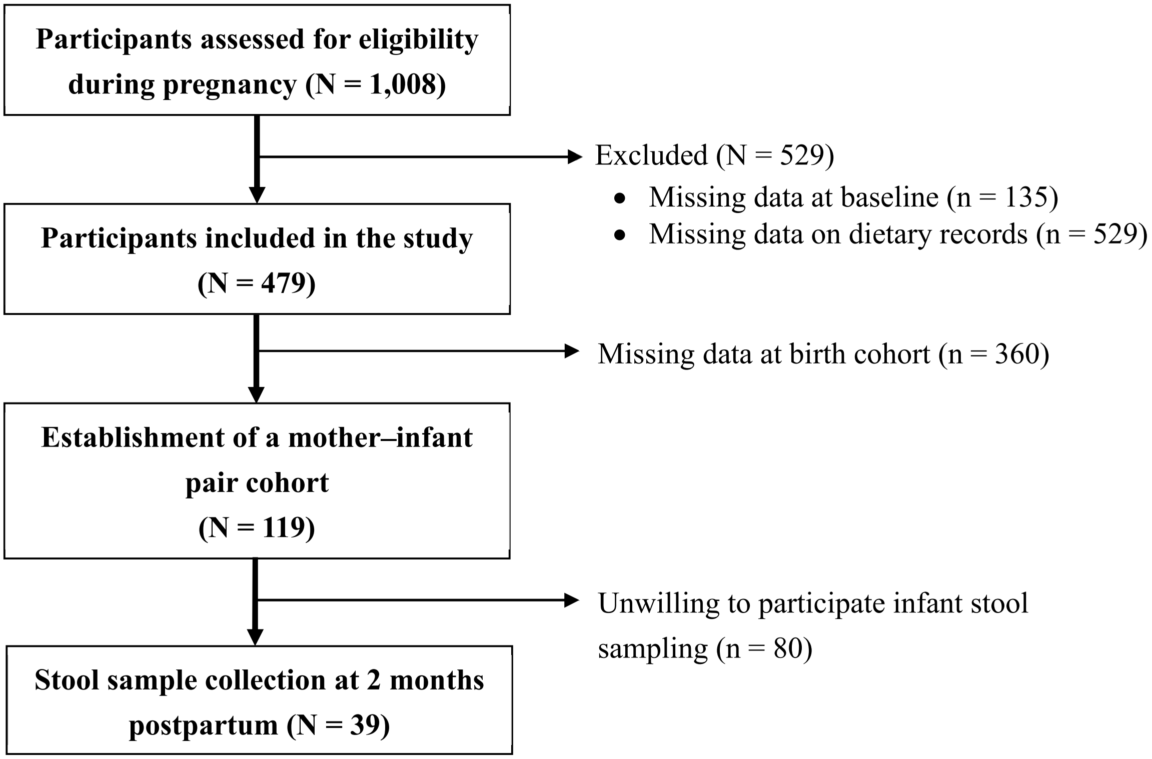

Figure S2. Alpha diversity of the infant gut microbiome according to maternal vegetable and fruit consumption during pregnancy

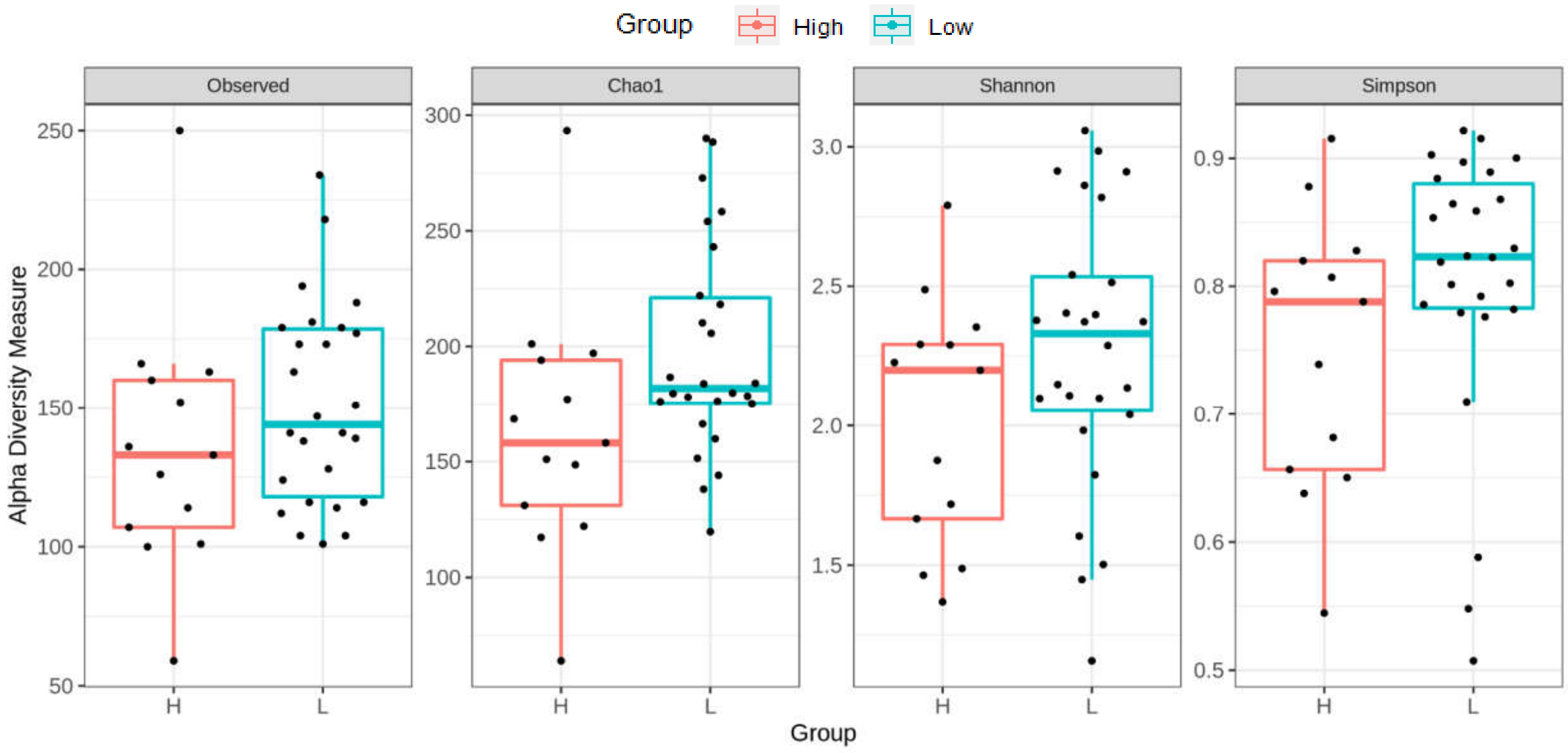

*High consumption was defined as one or more cups of fruits or vegetables per day.

Figure S3. Variations in the infant gut microbiome are not explained by other potential confounders

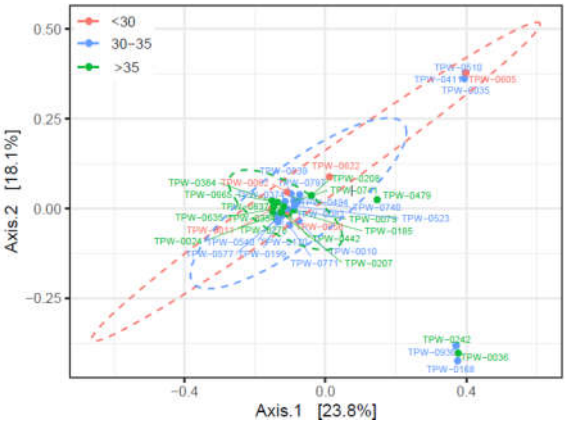

Adonis P = 0.43; Betadisper P = 0.69

(A) Age (years)

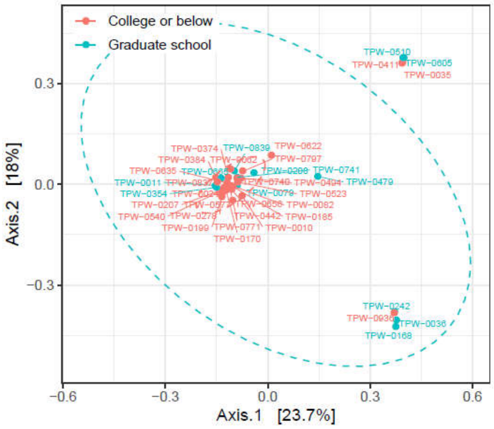

Adonis P = 0.05; Betadisper P = 0.06

(B) Education level

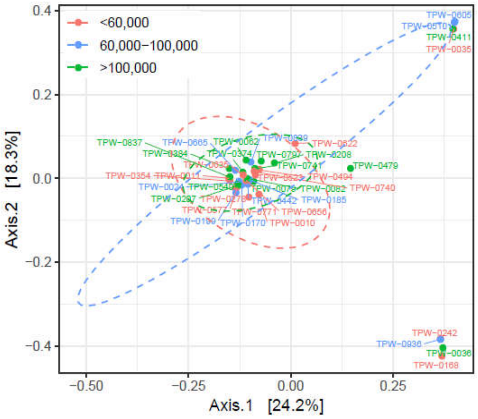

Adonis P = 0.85; Betadisper P = 0.96

(C) Family income (NT$)

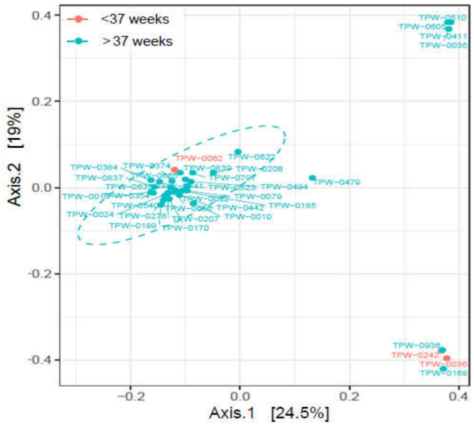

Adonis P = 0.05; Betadisper P = 0.25

(D) Gestational age (weeks)

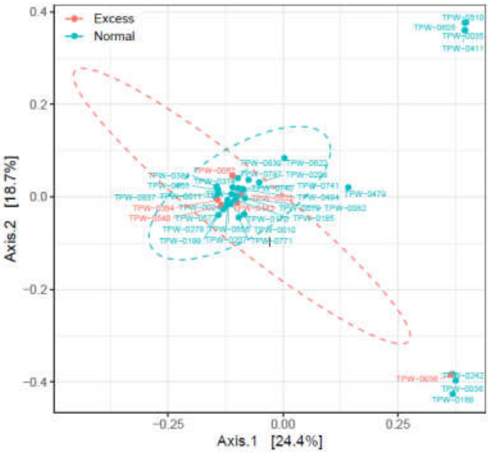

Adonis P = 0.70; Betadisper P = 0.33

(E) Excess gestational weight gain

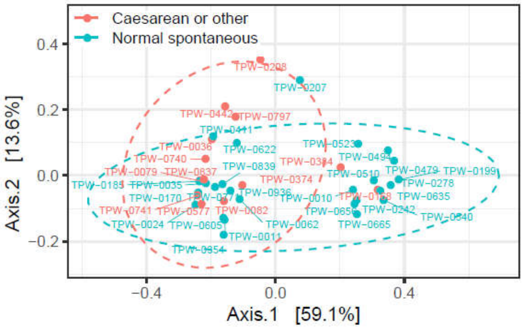

Adonis P = 0.33; Betadisper P = 0.26

(F) Delivery mode

Figure S3. Variations in the infant gut microbiome are not explained by other potential confounders (continued)

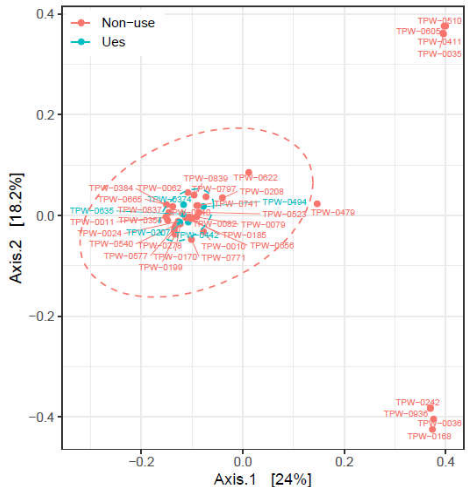

Adonis P = 0.36; Betadisper P = 0.06

(G) Antepartum antibiotics

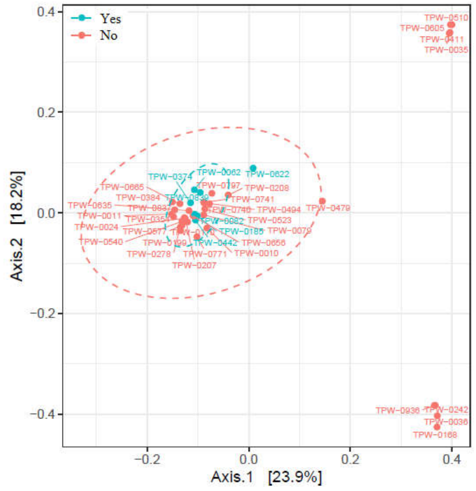

Adonis P = 0.28; Betadisper P = 0.01

(H) Group B streptococcus positive

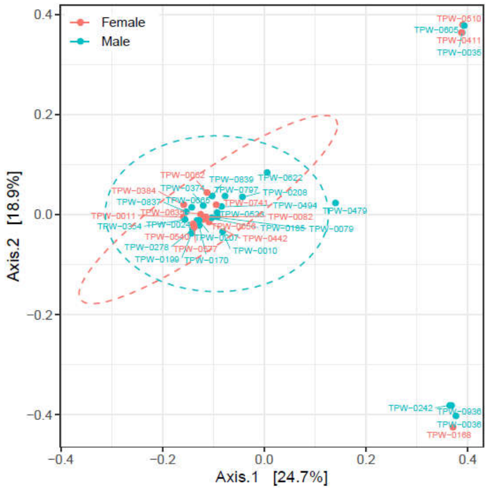

Adonis P = 0.95; Betadisper P = 0.57

(I) Neonatal sex

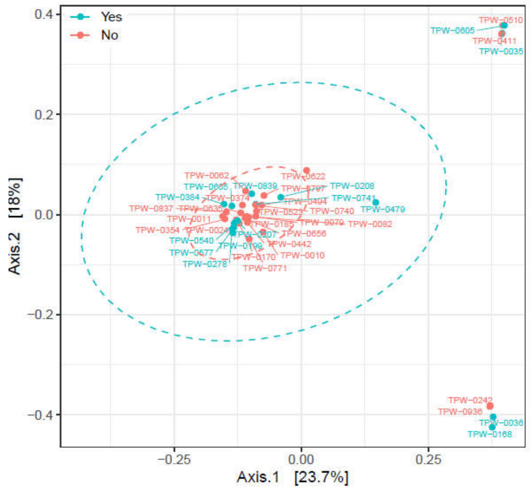

Adonis P = 0.77; Betadisper P = 0.16

(J) Breastfeeding

Figure S4. Relationship of intake of food from specific food groups to the abundance of specific gut microbiota

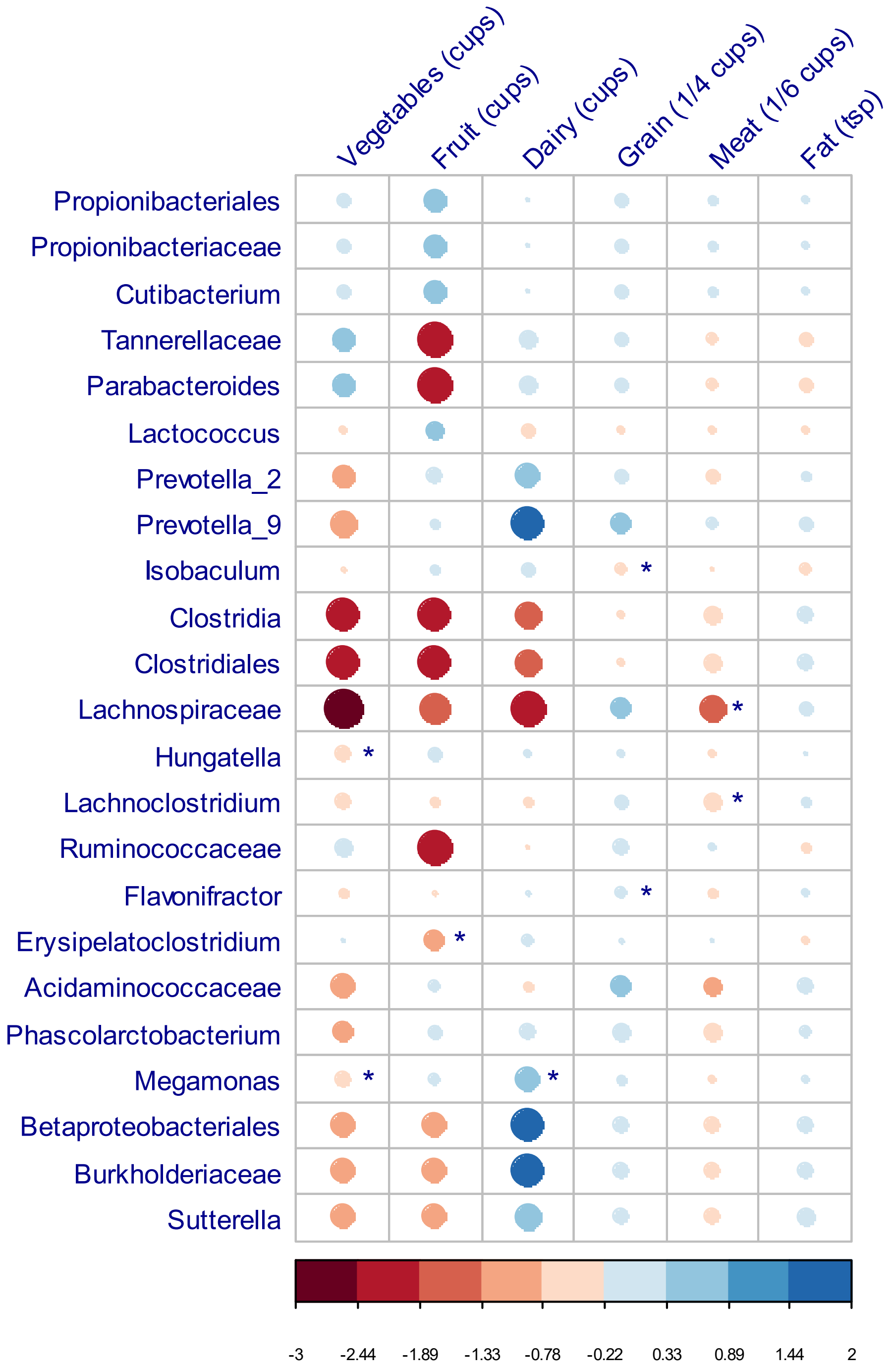

One cup = 240 mL; tbsp = tablespoon (5 mL).
*Asterisk indicates significant association
